# Epstein-Barr virus-associated lymphomas: biology, molecular genomics and precision oncology

**DOI:** 10.3389/fonc.2025.1677060

**Published:** 2025-11-24

**Authors:** Soumyadeep Datta, Ajay Gogia, Saumyaranjan Mallick

**Affiliations:** 1 Department of Medicine, All India Institute of Medical Sciences, New Delhi, India; 2 Department of Medical Oncology, Dr. B.R. Ambedkar - Institute Rotary Cancer Hospital, All India Institute of Medical Sciences, New Delhi, India; 3 Department of Pathology, All India Institute of Medical Sciences, New Delhi, India

**Keywords:** Epstein-Barr virus, lymphoid malignancies, lymphomas, immune checkpoint inhibitors, CAR-T

## Abstract

Epstein-Barr virus (EBV) is a ubiquitous human gamma-herpesvirus causally linked to a diverse spectrum of lymphoid malignancies. This review provides a comprehensive overview of EBV-associated lymphomas, encompassing their global epidemiology, the intricate pathogenesis driven by viral latency proteins and complex host immune interactions, and the varied clinical presentations of distinct subtypes. We delve into the detailed pathological features, molecular characteristics, and diagnostic strategies for classic Hodgkin lymphoma (cHL), Burkitt lymphoma (BL), diffuse large B-cell lymphoma (DLBCL), post-transplant lymphoproliferative disorder (PTLD), and extra-nodal NK/T-cell lymphoma, nasal type (ENKTL). Current subtype-specific treatment paradigms are critically evaluated, along with a thorough exploration of emerging therapeutic avenues, including novel immunotherapeutic approaches such as immune checkpoint inhibitors, adoptive cell therapies like EBV-specific cytotoxic T lymphocytes and chimeric antigen receptor T-cells (CAR-T), and targeted molecular therapies. Finally, we highlight the persistent challenges, critical knowledge gaps, and promising future prospects, including preventative and therapeutic vaccine strategies, aimed at optimizing diagnostic precision and improving long-term outcomes for patients afflicted with these heterogeneous and often aggressive diseases.

## Introduction

Epstein-Barr virus (EBV), a ubiquitous human gamma-herpesvirus holds a well-established and profound role in the development of several lymphoid and epithelial cancers (1). First identified in Burkitt lymphoma cells in 1964 by Epstein, Achong, and Barr (2), EBV has since been recognized as one of the most successful human pathogens infecting over 90% of the adult population globally (3). Following primary infection, typically asymptomatic in early childhood, EBV establishes a lifelong latent infection primarily within memory B-lymphocytes, where it persists in a dormant state (4). However, primary EBV infection during adolescence or early adulthood can manifest as infectious mononucleosis characterized by fever, pharyngitis, and lymphadenopathy, representing a robust host immune response to viral replication and B-cell proliferation (5).

Crucially, EBV is etiologically linked to a wide spectrum of lymphomas, ranging from well-recognized entities to less common variants (1). These include classic Hodgkin lymphoma (cHL), Burkitt lymphoma (BL), diffuse large B-cell lymphoma (DLBCL), post-transplant lymphoproliferative disorder (PTLD), and extra-nodal NK/T-cell lymphoma, nasal type (ENKTL) (1). These EBV-associated lymphomas can affect both immunocompetent and immunocompromised individuals, particularly in contexts of chronic immune suppression such as human immunodeficiency virus (HIV) infection, other primary or acquired immunodeficiencies, or post-transplantation (5). PTLD serves as a prime example of uncontrolled EBV-driven lymphocyte proliferation in the setting of impaired T-cell surveillance.

The oncogenic potential of EBV is mediated by a complex interplay of viral genes and host cellular pathways (6). During latency, EBV expresses a limited set of viral proteins including Epstein-Barr nuclear antigens (EBNAs), latent membrane proteins (LMPs), and non-coding ribonucleic acids (RNAs) such as EBV-encoded RNAs (EBERs) and micro-RNAs (miRNAs) (7). These viral products orchestrate a profound transformation of infected B-cells, promoting their proliferation, inhibiting apoptosis, and enabling immune evasion (8). Moreover, the specific type of latency program expressed; latency I, II, or III defines the pattern of viral gene expression and consequently dictates the precise type of lymphoma that develops (9). These distinct latency profiles not only drive the unique biological characteristics of each tumor but also significantly influence host immune surveillance mechanisms and ultimately the response to therapeutic interventions (9). Given the remarkable diversity in clinical behavior, histological presentation, molecular features, and treatment responses among EBV-associated lymphomas, there is a growing and urgent imperative for subtype-specific diagnostics, precise prognostication, and highly targeted therapeutic approaches. This comprehensive review aims to provide an in-depth overview of the pathobiology, detailed diagnostic methods, current treatment modalities, and prospective therapeutic strategies for EBV-associated lymphomas, highlighting key challenges and future directions in the field.

## Epstein-Barr virus: overview

EBV, officially designated as human herpesvirus 4 (HHV-4), is a double-stranded DNA virus belonging to the *Gammaherpes virinae* subfamily of the *Herpesviridae* family. Its approximately 172-kilobase pair genome encodes over 80 genes. Following primary infection, which often occurs through saliva, EBV preferentially infects B-lymphocytes, establishing a lifelong latent infection characterized by its ability to immortalize these cells *in vitro* (10). This persistence is maintained within memory B-cells for the host’s lifetime, where the virus remains largely dormant but can periodically reactivate under certain physiological or immunosuppressive conditions, leading to lytic replication and viral shedding (10).

**Figure 1:**
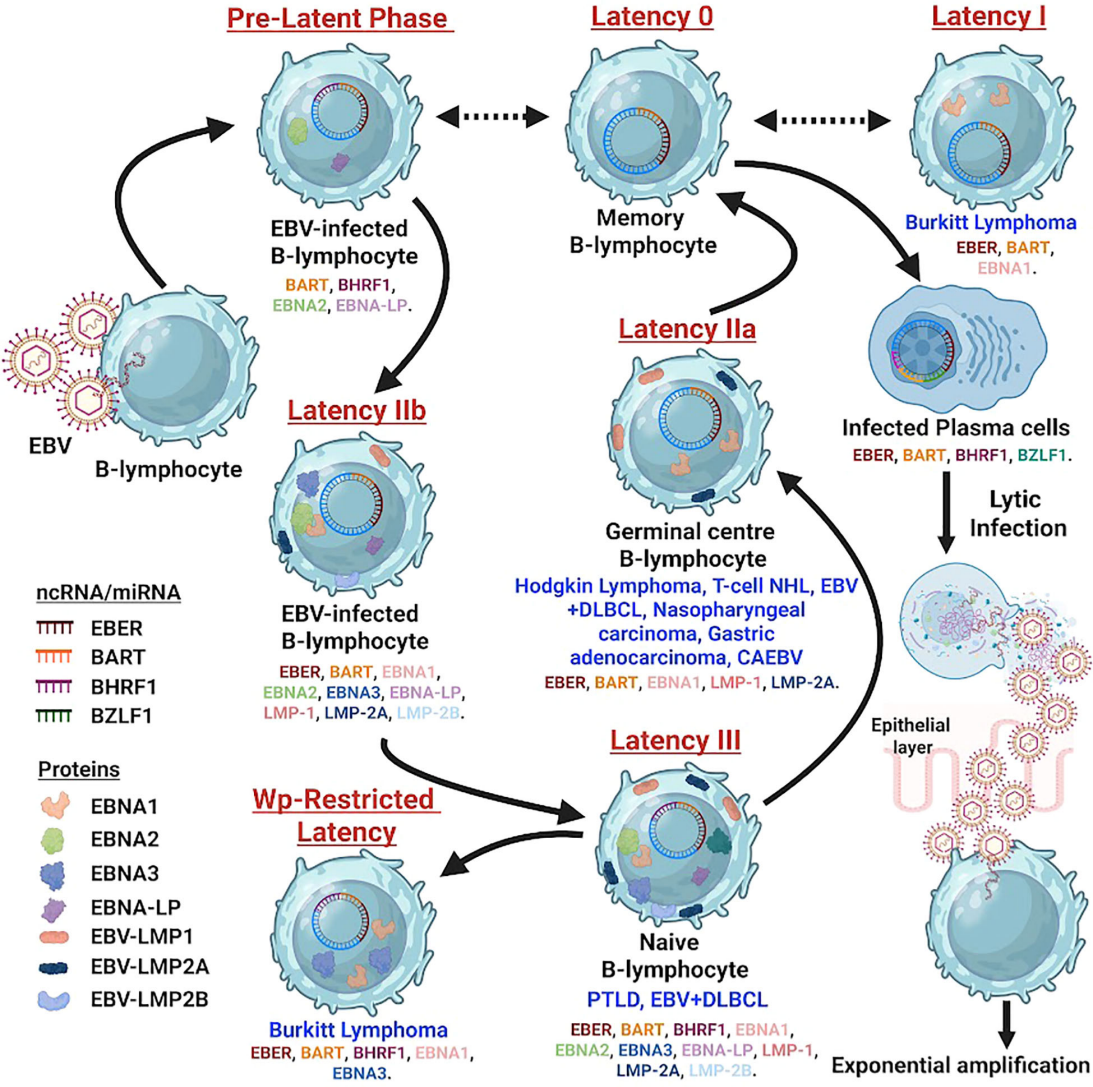
Schematic diagram depicting the EBV infection cycle in B-cells, beginning with viral entry through the oropharyngeal epithelium and progression from lytic replication to multiple latency states (0, I, IIa, IIb, III). Color-coded symbols represent EBV proteins and non-coding RNAs, including EBNA1–6, LMP1, LMP2A/2B, EBERs, BHRF1, BARTs, and EBV miRNAs. Arrows show movement of infected cells from naïve B-cells through germinal centers into memory or lymphoblast stages. Each latency phase is linked to specific diseases such as Burkitt lymphoma, Hodgkin lymphoma, nasopharyngeal carcinoma, CAEBV, PTLD, and EBV-positive DLBCL, with lytic reactivation shown during plasma cell differentiation.

A hallmark of EBV’s interaction with the host cell is its ability to establish distinct latency programs, each characterized by a specific pattern of viral gene expression (**Figure 1**). These programs dictate the cellular tropism, oncogenic potential, and clinical manifestation of EBV-associated diseases.

Latency I (latency program): This is the most restricted form of latency, characterized by the expression of only Epstein-Barr nuclear antigen 1 (EBNA1) and often the non-coding EBERs and BamHI-A rightward transcripts (BARTs) miRNAs (9, 11). EBNA1 is essential for the replication and segregation of the viral episome during cell division, ensuring the persistence of the viral genome in daughter cells. This latency type is predominantly observed in endemic BL (9).

Latency II (default program): In addition to EBNA1, EBERs, and BARTs, latency II involves the expression of latent membrane proteins 1 (LMP1) and 2A/2B (LMP2A/2B) (9, 12). LMP1 is a potent oncogene that mimics a constitutively active CD40 receptor, activating critical signaling pathways like nuclear factor-kappa B (NF-κB), mitogen-activated protein kinase (MAPK), and phosphatidylinositol 3-kinase (PI3K)/AKT (8). LMP2A mimics the B-cell receptor, maintaining B-cell survival in the absence of exogenous stimulation (13). This latency type is characteristic of cHL, nasopharyngeal carcinoma, and ENKTL (9, 12).

Latency III (growth program): This is the most comprehensive latency program, expressing all six EBNAs (EBNA1, 2, 3A, 3B, 3C, LP), LMP1, LMP2A/2B, EBERs, and BARTs (9, 12). EBNA2 is a transcriptional activator crucial for driving the expression of other latent genes, including LMP1 and LMP2, and several host genes (14). This broad viral gene expression promotes the robust proliferation and transformation of B-cells. Latency III is typically observed in PTLD and some cases of DLBCL (9, 12).

Beyond the protein-coding genes, EBV also expresses non-coding RNAs, such as the abundant EBV-encoded RNAs (EBERs) and a complex repertoire of miRNAs derived from BARTs (7). EBERs are believed to contribute to immune evasion and cellular transformation, while BARTs miRNAs play a critical role in modulating host gene expression, impacting cellular proliferation, apoptosis, and immune surveillance (7, 15). Understanding these intricate latency programs and the functions of their associated viral products is fundamental to deciphering the diverse oncogenic mechanisms and clinical manifestations of EBV-associated lymphomas.

## Pathogenesis of EBV-associated lymphomas

The pathogenesis of EBV-associated lymphomagenesis is a multifaceted process resulting from complex interactions between viral oncogenes, the host immune system, and contributing environmental cofactors. EBV’s ability to establish latent infection and express specific viral proteins is central to its oncogenic potential.

### Latent protein activity

The latent proteins of EBV are key drivers of lymphomagenesis. LMP1 is arguably the most critical oncoprotein (8). It is a functional analogue of a constitutively active CD40 receptor, an important co-stimulatory molecule in B-cell activation. LMP1 activates several key signaling pathways, including NF-κB, activator protein 1 (AP-1), Janus kinase/signal transducer and activator of transcription (JAK/STAT), and the PI3K/AKT pathway (8, 16). Activation of these pathways promotes cell proliferation, enhances survival by inhibiting apoptosis, and upregulates the expression of adhesion molecules and cytokines, thereby fostering an environment conducive to tumor growth. LMP2A mimics the signaling of a constitutively active B-cell receptor, contributing to cell survival and proliferation in the absence of antigen stimulation (13). It can also inhibit tyrosine kinase Lyn and suppress B-cell receptor-mediated signaling, potentially preventing infected B-cells from undergoing differentiation or apoptosis (17).

### Immune evasion

EBV has evolved sophisticated mechanisms to evade host immune surveillance, which is critical for its persistence and for the survival of transformed cells. One significant mechanism involves the downregulation of major histocompatibility complex (MHC) class I and II molecules on the surface of infected cells (18). This prevents effective presentation of viral antigens to cytotoxic T lymphocytes (CTLs), thereby allowing EBV-infected cells to escape immune recognition and destruction. Furthermore, EBV produces a viral interleukin-10 (vIL-10) homolog, which suppresses T-cell responses and inhibits the production of pro-inflammatory cytokines, further contributing to local immunosuppression within the tumor microenvironment (18, 19). The expression of high levels of programmed death-ligand 1 (PD-L1) by EBV-infected tumor cells is another critical immune evasion strategy, leading to T-cell exhaustion and an inability of the immune system to clear the malignant cells (20, 21).

### Genomic instability

While EBV itself does not directly cause gene mutations in the same way as some other oncogenic viruses, its chronic presence and the activity of its latent proteins can indirectly contribute to genomic instability. For instance, EBV infection can induce the expression of activation-induced cytidine deaminase (AID) in B-cells (22). AID is an enzyme crucial for somatic hypermutation and class switch recombination in normal B-cell development. However, dysregulated AID activity in EBV-infected cells can lead to off-target mutations and chromosomal translocations, such as the characteristic t(8;14) translocation that juxtaposes the MYC oncogene to immunoglobulin heavy chain (IgH) loci, leading to its overexpression in BL (22, 23). This highlights a mechanism by which chronic viral presence can subvert normal cellular processes to promote oncogenesis.

### Microenvironmental factors

The tumor microenvironment plays a crucial role in the development and progression of EBV-associated lymphomas. EBV-infected tumor cells can recruit and reprogram various stromal and immune cells, creating an immunosuppressive milieu that supports tumor growth and progression (24). This includes the presence of regulatory T-cells (Tregs), which suppress anti-tumor immune responses and tumor-associated macrophages (TAMs), which can promote angiogenesis and tumor cell proliferation (24, 25). Cytokines and chemokines secreted by both tumor cells and surrounding stromal cells further contribute to this pro-tumorigenic and immunosuppressive environment, impairing the efficacy of endogenous anti-tumor immunity. For example, LMP1 can induce the production of various chemokines and cytokines, including IL-6 and TNF-α, which can contribute to the inflammatory and immunosuppressive microenvironment (16).

## Classification and types of EBV-associated lymphomas

EBV is implicated in a diverse array of lymphoid malignancies, each characterized by distinct clinical, pathological, and molecular features. The classification of these lymphomas often considers the predominant cell type, anatomical site, and the specific EBV latency program expressed.

### Classic Hodgkin lymphoma

EBV is detectable in 20–50% of cHL cases globally, with higher rates observed in pediatric, elderly, and HIV-infected patients, as well as in developing countries (26, 27). The characteristic malignant cells in cHL, Reed-Sternberg (RS) cells and their variants, are consistently of B-cell origin and express the EBV latency II program (9, 26). LMP1 plays a crucial role in the survival and proliferation of RS cells. EBV-positive cHL often presents with specific histological subtypes, notably mixed cellularity and lymphocyte-depleted HL, and may have a different clinical course compared to EBV-negative cases, potentially responding more favorably to certain immunotherapies (27, 28).

### Burkitt lymphoma

BL is an aggressive B-cell non-Hodgkin lymphoma characterized by high proliferation and typically a MYC gene translocation (23). EBV association varies by epidemiological form:

Endemic BL: Highly prevalent in equatorial Africa and closely associated with *Plasmodium falciparum* malaria infection, which is thought to impair immune control of EBV (29). Endemic BL is almost universally (95–100%) EBV-positive and expresses the latency I program (9, 29).

Sporadic BL: Occurs worldwide, less common than endemic form, and shows EBV positivity in only 10–20% of cases (29).

Immunodeficiency-associated BL: Seen in immunocompromised individuals (e.g., HIV, post-transplant), with high rates of EBV positivity; often latency III (30). Regardless of EBV status, all forms of BL are characterized by the t(8;14) translocation or variants [t(2;8), t(8;22)], leading to constitutive activation of the MYC oncogene (23).

### Diffuse large B-cell lymphoma

EBV-positive DLBCL, not otherwise specified (NOS), is recognized as a distinct entity in the World Health Organization (WHO) classification (31). It is predominantly observed in elderly individuals (typically >50 years) and those with immunosuppression, though it can occur in immunocompetent younger patients (32). These lymphomas frequently express either the latency II or III profile (32). EBV-positive DLBCL generally exhibits a poorer prognosis compared to EBV-negative cases, characterized by more aggressive clinical features, higher rates of central nervous system (CNS) involvement, and often resistance to standard rituximab-cyclophosphamide, doxorubicin, vincristine, prednisone (R-CHOP) chemotherapy (33, 34). The presence of EBV in these tumors suggests a unique biology that warrants specific therapeutic considerations.

### Post-transplant lymphoproliferative disorder

PTLD represents a heterogeneous group of lymphoid proliferations that arise in the setting of iatrogenic immunosuppression following solid organ or hematopoietic stem cell transplantation (35). The vast majority (>80%) of PTLD cases are EBV-positive, particularly those occurring early after transplantation (35). The pathogenesis is driven by the uncontrolled proliferation of EBV-infected B-cells due to impaired T-cell immune surveillance (36). PTLD spans a broad spectrum of morphological presentations, from benign polyclonal plasmacytic hyperplasia to aggressive monomorphic lymphomas resembling DLBCL or BL. These lymphomas typically express the latency III profile (9, 35). Reduction of immunosuppression is often the first line of management, leading to remission in a significant proportion of cases through restoration of EBV-specific T-cell immunity (36).

In the 5^th^ edition of the WHO classification of hemato-lymphoid neoplasms, a significant change has been introduced in the categorization of immunodeficiency-related lymphoproliferative disorders (LPDs) (37). In the 4^th^ edition, these disorders were classified under four distinct headings; post-transplant, HIV-associated, primary immunodeficiency-related, and iatrogenic immunosuppression-related. The current classification consolidates these entities into a unified framework, presenting a three-part diagnostic approach that includes; histopathological features, associated viral agents, and underlying clinical context (37). Under the histological features, the three major categories are; hyperplasia, lymphoproliferative disorder of varied malignant potential, and lymphomas. Hyperplasia includes benign conditions like follicular hyperplasia, plasmacytic hyperplasia, infectious mononucleosis, HHV-associated Castleman disease, and other hyperplasia and involutions. The second category includes polymorphic LPDs, and muco-cutaneous ulcer, while the last category includes lymphomas as per the main classification. This three-tier diagnostic system includes all the entities and their related conditions, hence, will be helpful for clinical decision making and excluding unnecessary repetition of similar morphological entities of lymphomas or LPDs in different clinical conditions.

### Extra-nodal NK/T-cell lymphoma, nasal type

ENKTL is a rare, aggressive lymphoma with a strong geographical predilection for East Asia, Latin America, and other regions, accounting for a significant proportion of lymphomas in these areas (38). It is virtually 100% associated with EBV, with tumor cells expressing the EBV latency II program (9, 38). Clinically, ENKTL often presents as a destructive lesion in the upper aerodigestive tract (nasal cavity, nasopharynx, palate), leading to symptoms like nasal obstruction, epistaxis, and facial swelling (39). However, extra-nasal involvement can occur, affecting the skin, gastrointestinal tract, or testis, and is associated with a poorer prognosis (39). ENKTL is known for its aggressive nature and resistance to conventional anthracycline-based chemotherapy regimens due to the high expression of p-glycoprotein (MDR1) (40).

## Diagnosis and biomarkers

**Figure 2:**
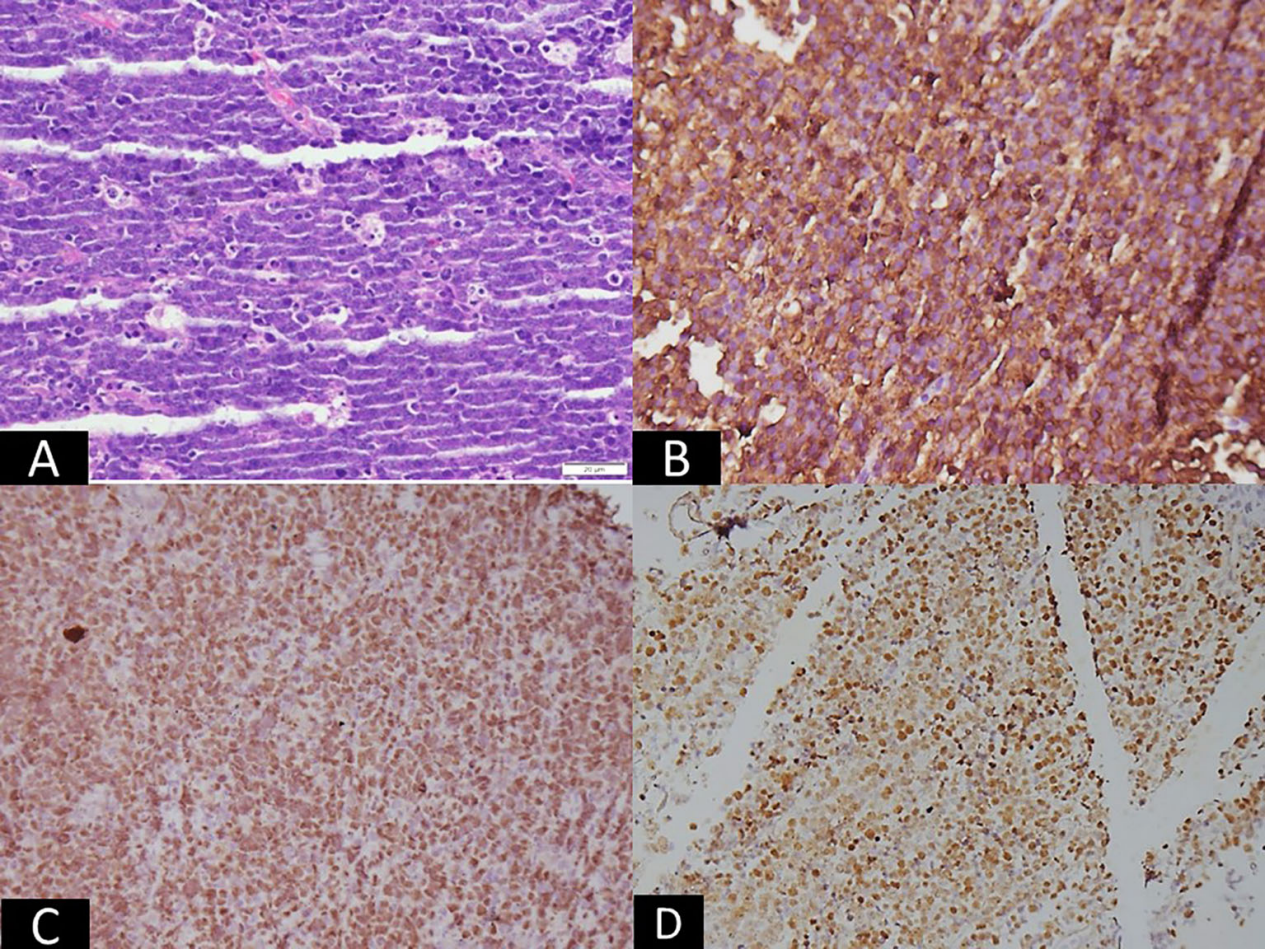
Microscopic panel showing intermediate-sized atypical lymphoid cells arranged in sheets with numerous tingible-body macrophages, creating a starry-sky pattern (H&E, ×200). Adjacent immunohistochemistry images show strong membranous staining for CD20 (×100) and nuclear positivity for c-MYC (×100). An in-situ hybridization image demonstrates strong nuclear EBER signals (×200). The combined panels visually indicate Burkitt lymphoma, corresponding to EBV latency I, characterized by monomorphic lymphoid cells and EBER positivity.

**Figure 3:**
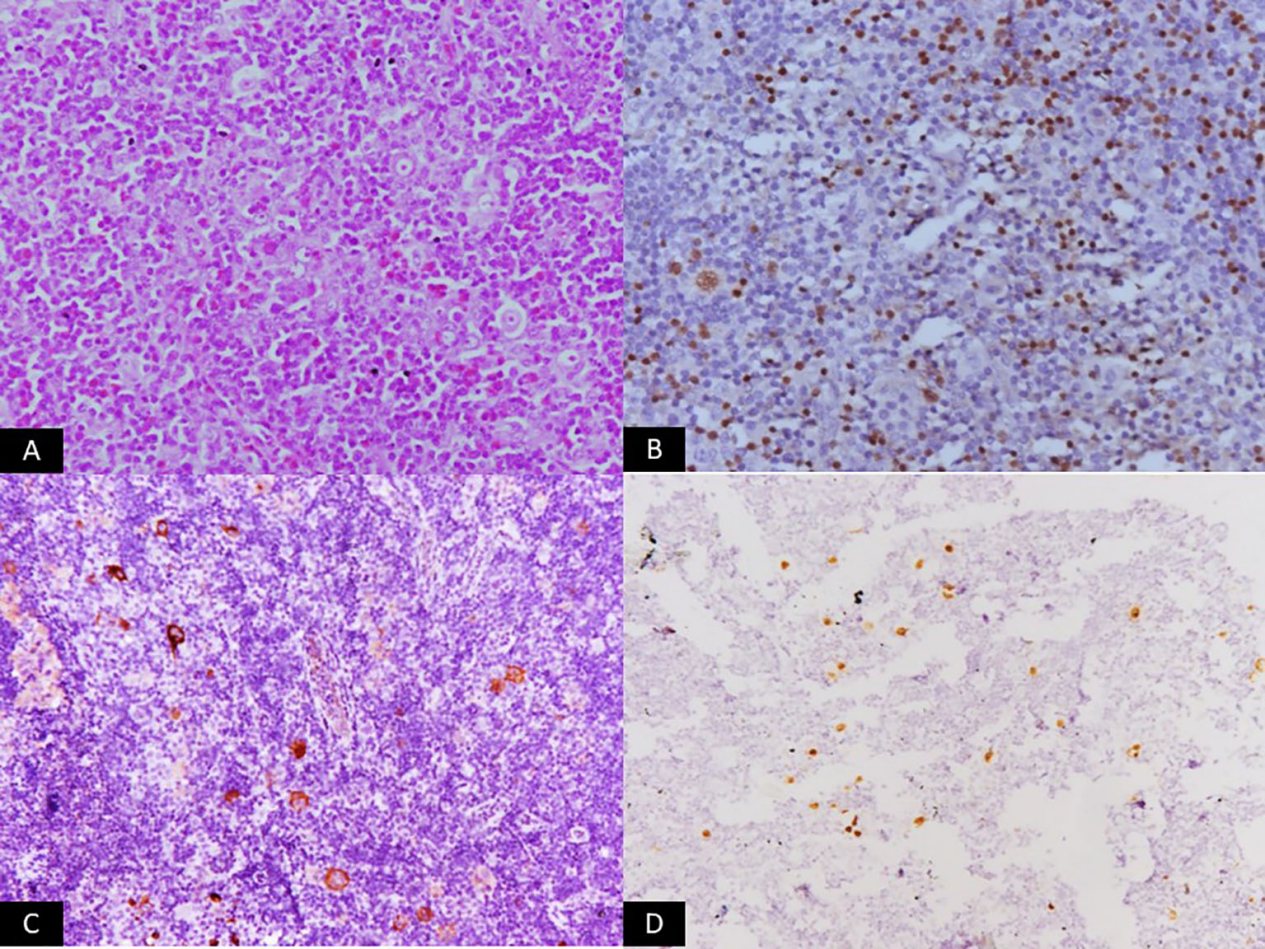
Microscopic panel showing a polymorphous infiltrate composed of small lymphocytes, eosinophils, histiocytes, and scattered classic Reed–Sternberg cells (H&E, ×200). Additional immunohistochemistry panels display dim nuclear staining for PAX5 (×200) and membranous/cytoplasmic positivity for EBV-LMP1 (×200). An in-situ hybridization panel shows strong nuclear EBER signals (×200). The set of images visually represents classic Hodgkin lymphoma associated with EBV latency II.

**Figure 4:**
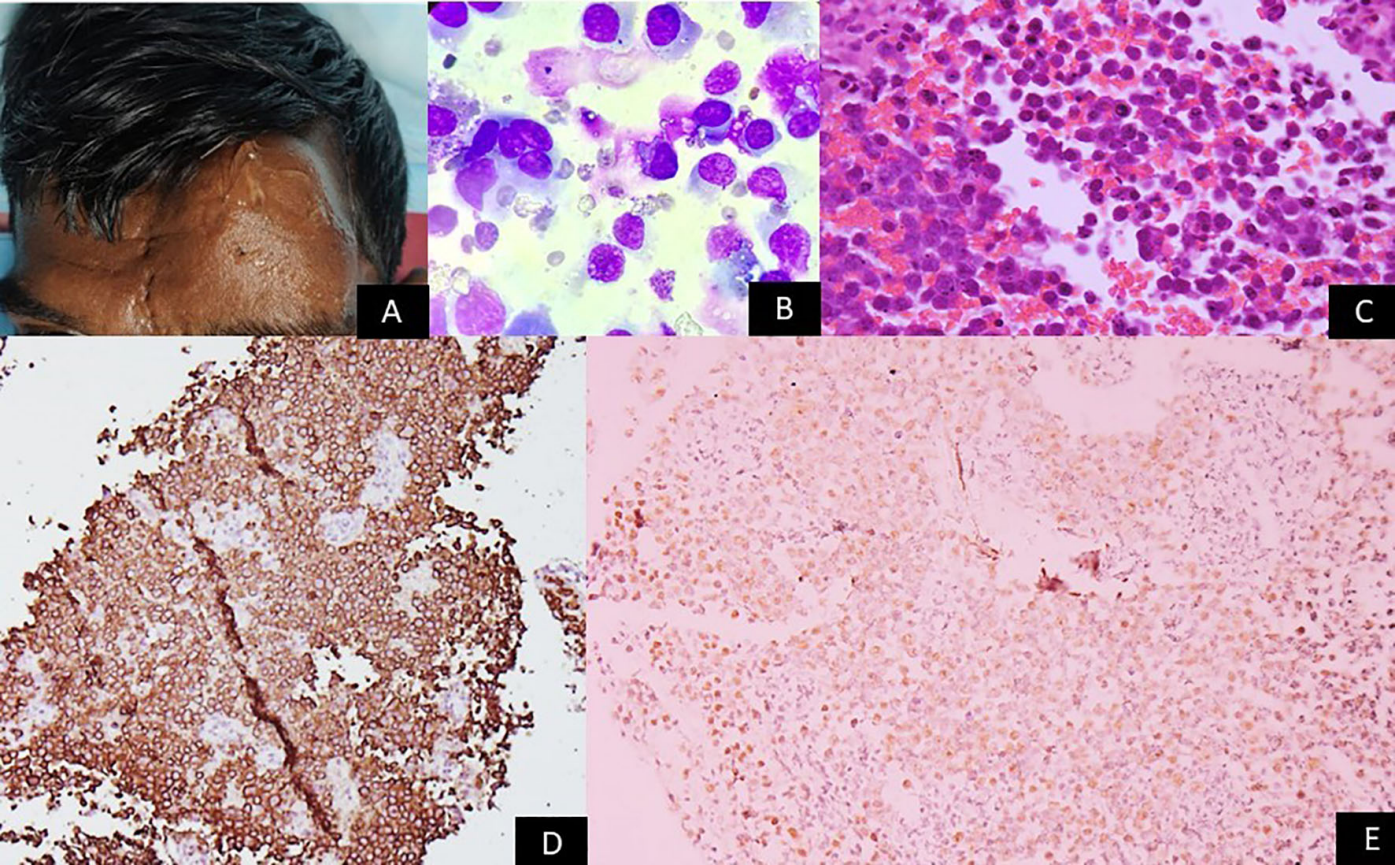
Gross image showing a person living with HIV with a large nodular swelling on the forehead. Microscopy panels show sheets of large atypical lymphoid cells with moderate cytoplasm (Giemsa ×200 and H&E ×200). Immunohistochemistry reveals strong CD138 membranous staining (×100), and in-situ hybridization shows nuclear EBER positivity (×200). The images collectively depict features consistent with plasmablastic lymphoma, corresponding to EBV latency III.

Accurate diagnosis and prognostication of EBV-associated lymphomas rely on a comprehensive approach integrating histopathology, immunophenotyping, and molecular techniques for EBV detection (**Figures 2**–**4**).

### Histology and immunohistochemistry

The initial diagnosis of lymphoma is based on histological examination of tissue biopsy. Morphological features vary widely across different EBV-associated lymphoma subtypes. For instance, cHL is characterized by the presence of large, often binucleated RS cells, while BL exhibits a monotonous proliferation of medium-sized lymphoid cells with a ‘starry-sky’ pattern (23, 41). Immunophenotyping using IHC is crucial for lineage assignment and differentiation from other lymphoid neoplasms. Common markers include CD20 for B-cell lymphomas, CD3 for T-cell lymphomas, and CD30 and CD15 for cHL. For NK/T-cell lymphomas, markers like CD2, cytoplasmic CD3, CD56, and cytotoxic granules (granzyme B, perforin, TIA-1) are typically positive (39).

### EBV detection methods

Direct detection of EBV within tumor cells is critical for establishing an EBV-associated lymphoma diagnosis.

EBV-encoded RNA (EBER) *in-situ* hybridization (ISH): EBER ISH is considered the gold standard for detecting EBV in tissue sections due to its high sensitivity and specificity (42). EBERs are small, non-coding RNAs expressed at high copy numbers in virtually all EBV latency programs, making them an excellent molecular marker for the presence of EBV-infected cells (42). A positive EBER ISH confirms the presence of EBV in the malignant cells.

IHC for latent proteins: IHC can detect the expression of specific EBV latent proteins, particularly LMP1 and EBNA2. LMP1 is commonly expressed in latency II (cHL, ENKTL) and latency III (PTLD), while EBNA2 is uniquely expressed in latency III (12). IHC for these proteins can provide insights into the specific EBV latency program, aiding in classification and understanding of pathogenesis.

Polymerase chain reaction (PCR) and quantitative PCR (qPCR) for EBV DNA: PCR-based methods can detect EBV DNA in tissue or circulating cell-free DNA (cfDNA) in plasma (43). qPCR allows for the quantification of EBV DNA load, which is particularly useful for diagnosis and monitoring disease activity in conditions like PTLD and ENKTL (43, 44). Elevated pre-treatment EBV DNA levels often correlate with increased tumor burden and poorer prognosis, and a reduction in viral load post-treatment can indicate therapeutic response (44).

### Emerging biomarkers

Beyond EBV detection, several emerging biomarkers are being investigated for their prognostic or therapeutic implications.

Programmed death-ligand 1 (PD-L1): High expression of PD-L1 is frequently observed in EBV-associated lymphomas, particularly cHL and ENKTL, often driven by EBV-mediated signaling (e.g., LMP1 activation of JAK/STAT) (20, 21). PD-L1 expression can serve as a predictive biomarker for response to immune checkpoint inhibitors. PD-L1 expression in EBV-associated lymphomas is assessed on formalin-fixed paraffin-embedded tissue using IHC, with parallel EBV confirmation by EBER ISH. Tumor and immune cell membranous staining are evaluated, and expression quantified using tumor proportion score (TPS), combined positive score (CPS), or H-score. Results are correlated with EBV status, as EBV-positive lymphomas frequently exhibit PD-L1 overexpression. Appropriate antibody clones, validated platforms, and internal controls ensure reliable assessment for prognostic and therapeutic interpretation.

EBV-Encoded micro-RNAs (miRNAs): EBV expresses its own set of miRNAs (BARTs), which can be detected in tumor tissue and plasma. Their levels may serve as prognostic indicators or targets for novel therapies.

Host genetic signatures: Research is exploring host genetic alterations and gene expression profiles that interact with EBV infection to drive lymphomagenesis, potentially revealing new therapeutic targets.

## Current treatment strategies

Treatment strategies for EBV-associated lymphomas are largely dictated by the specific lymphoma subtype, disease stage, patient’s performance status, and prior treatment history. While EBV positivity can influence prognosis in certain settings, its direct impact on first-line treatment choice varies.

### cHL

For early-stage cHL, combined modality therapy involving chemotherapy (e.g., ABVD: doxorubicin, bleomycin, vinblastine, dacarbazine) followed by involved-site radiation therapy (ISRT) is standard. For advanced-stage disease, ABVD remains a common regimen, though dose-escalated BEACOPP (bleomycin, etoposide, doxorubicin, cyclophosphamide, vincristine, procarbazine, prednisone) is also used for high-risk patients (28, 45). While EBV status does not currently dictate initial treatment selection for cHL, EBV-positive cHL has been shown to exhibit a higher expression of PD-L1, making it more amenable to immune checkpoint blockade in relapsed or refractory settings (28, 46).

### BL

This being an extremely aggressive lymphoma, requires rapid initiation of intensive, short-duration multi-agent chemotherapy regimens to achieve cure. Common regimens include CODOX-M/IVAC (cyclophosphamide, vincristine, doxorubicin, methotrexate, ifosfamide, etoposide, cytarabine) or DA-EPOCH-R (dose-adjusted etoposide, prednisone, vincristine, cyclophosphamide, doxorubicin, rituximab) (47). Due to the high risk of CNS involvement, mandatory CNS prophylaxis with intrathecal chemotherapy is crucial. While EBV positivity is a defining feature of endemic BL, its presence does not generally alter the chemotherapy regimen for BL, as the underlying biology is driven by MYC deregulation regardless of EBV status (47).

### EBV-positive DLBCL, NOS

For newly diagnosed cases, the standard first-line treatment is R-CHOP, similar to EBV-negative DLBCL. However, given its generally poorer prognosis, particularly in elderly patients, there is ongoing research into more intensified or novel approaches for this subgroup (33, 34). This includes exploring the addition of agents like bortezomib, lenalidomide, or immune checkpoint inhibitors in clinical trials, but these are not yet standard of care.

### PTLD

Management of PTLD is highly individualized and depends on the type of transplant, disease extent, and specific PTLD subtype. The cornerstone of treatment for most EBV-positive PTLDs is reduction of immunosuppression (RIS) (36). RIS often leads to disease regression by restoring EBV-specific T-cell immunity against the proliferating B-cells. For patients who do not respond to RIS or have aggressive disease, further therapies include rituximab for CD20-positive B-cell PTLDs, and multi-agent chemotherapy regimens (e.g., CHOP or R-CHOP) particularly for monomorphic PTLDs resembling DLBCL (36).

### ENKTL

Aggressive nature and inherent resistance to anthracyclines are bottlenecks in treatment of this lymphoma (40). Standard treatment often involves non-anthracycline-based regimens combined with radiation therapy, especially for localized disease. Regimens incorporating L-asparaginase (e.g., SMILE: dexamethasone, methotrexate, ifosfamide, L-asparaginase, etoposide) have demonstrated superior outcomes (40). For advanced or relapsed/refractory ENKTL, novel agents and immunotherapies are actively being investigated, given the high expression of PD-L1 in these tumors (39, 46).

**Table 1** summarizes key clinical trials evaluating treatment in EBV-associated lymphomas.

**Table 1:** Clinical trials evaluating treatment in EBV-associated lymphomas.

Trial/study	Disease subtype and setting	Intervention	Phase	Sample size and population	Efficacy outcomes	Key safety findings
IVORY (54179060LYM2003) (48)	Newly diagnosed EBV^+^ DLBCL	Ibrutinib + R-CHOP	II	n = 24; median age 58 yrs	ORR 66.7%; CR 67%; CR in <65 yrs: 87.5% *vs* ≥65 yrs: 25%	Serious infections in elderly; 4 treatment-related deaths
NAVAL-1 (Stage 1) (49)	R/R EBV^+^ PTCL	Nanatinostat (class I HDAC inhibitor) + Valganciclovir	II	n = 20; 10 in each arm	Combo arm (ITT): ORR 50%, CR 20%; evaluable: ORR 71%, CR 29%	Grade ≥3 hematologic/GI AEs; 1 fatal sepsis
NAVAL-1 (Stage 1–2) (49, 50)	R/R EBV^+^ PTCL (expanded cohort)	Nanatinostat + Valganciclovir	II	n = 21 in combo arm	ITT: ORR 33%, CR 19%; evaluable: ORR 41%, CR 24%	Grade 5 sepsis in 1 patient
NAVAL-1 Phase 1b/2	R/R EBV^+^ lymphomas (all types)	Nanatinostat + Valganciclovir	I/II	n = 55	ORR 40%; CR 19%; median DOR: 10.4 months	Mostly grade 1–2 hematologic and GI events
Tabelecleucel (Ebvallo) (51)	EBV^+^ PTLD post-HSCT/SOT	Allogeneic EBV-specific T-cells (CAR-T)	III	Pivotal trial; multinational	ORR ~50–60% in PTLD	Well tolerated, low GVHD risk
Nivolumab/Pembrolizumab trials (52, 53)	EBV^+^ DLBCL, HL, ENKTL, PTLD	Checkpoint inhibitors	I/II	Early-phase; multiple studies	Promising responses in ENKTL and PTLD	Immune-related AEs typical of class
Brentuximab Vedotin trial (54)	CD30^+^ EBV^+^ lymphomas (incl. PTCL, HL)	BV monotherapy	II	Ongoing; n < 30	Interim data: ORR > 20% expected	TBD
Baltaleucel-T trial (55)	R/R ENKTL	Autologous EBV-specific T-cells (CAR-T)	II	Small cohort	ORR 50%, CR 30%; some durable responses	Favorable; minimal toxicity
Third-party EBV-CTLs (NCT01498484) (56)	EBV^+^ LPD/lymphoma post-transplant	EBV-CTLs (banked)	II	n = 33	CR/PR ≥ 58%; durable responses	Minimal GVHD

## Emerging therapies and future prospects

The unique biology of EBV-associated lymphomas, particularly their dependence on viral proteins and interactions with the host immune system, offers distinct opportunities for novel therapeutic interventions.

### Immune checkpoint inhibitors

Immune checkpoint inhibitors (ICIs), particularly those targeting the PD-1/PD-L1 pathway, have transformed the therapeutic landscape of oncology by harnessing the host immune system to fight cancer. Their efficacy in Epstein–Barr virus (EBV)-associated lymphomas is increasingly recognized due to the virus’s intrinsic role in immune evasion. EBV-infected tumor cells, particularly in classical Hodgkin lymphoma (cHL) and extranodal NK/T-cell lymphoma (ENKTL), often show marked overexpression of PD-L1 on their surface, driven by EBV-encoded proteins such as LMP1 and EBNA2, as well as by genetic alterations in the PD-L1/PD-L2 locus (20, 21). This PD-L1 upregulation suppresses cytotoxic T-cell function by engaging PD-1 receptors on T-cells, leading to T-cell exhaustion and impaired anti-tumor surveillance.

Blocking this interaction with anti–PD-1 antibodies such as nivolumab and pembrolizumab reinvigorates exhausted T-cells, restoring cytokine production and cytotoxic activity against tumor cells. Clinical trials have demonstrated remarkable response rates, particularly in relapsed or refractory cHL and ENKTL, where durable remissions have been observed even after multiple prior therapies (46, 57, 58). Furthermore, ICIs have shown favorable safety profiles, making them suitable for heavily pretreated or frail patients. Current research is exploring their use in earlier lines of therapy, in combination with chemotherapy or radiotherapy, or as consolidation after remission, with the goal of achieving deeper and more sustained responses through synergistic immune activation.

### EBV-specific CTLs

Adoptive transfer of ex vivo–expanded EBV-specific CTLs represents one of the most precise and biologically rational forms of immunotherapy in EBV-driven malignancies (59). This approach utilizes the natural immune defense against EBV by isolating T-cells capable of recognizing EBV antigens, expanding them outside the body, and then re-infusing them into the patient. The CTLs may be donor-derived, sourced from the transplant donor, third-party donors, or autologous (patient-derived) T-cells that are primed and selected for EBV antigen recognition.

These EBV-specific CTLs selectively target infected or transformed cells expressing latent viral antigens such as LMP1, LMP2, or EBNA proteins, thereby sparing healthy tissue. The therapy has achieved notable clinical success in PTLD, where it has induced durable and sometimes complete remissions (57, 60). The safety profile is also favorable, with minimal risk of graft-versus-host disease (GVHD) when appropriately matched.

Beyond PTLD, research is ongoing to extend this therapy to other EBV-associated cancers, including nasopharyngeal carcinoma and certain lymphomas with latency II or III expression profiles. However, certain challenges remain. tumors with restricted latency programs (e.g., expressing only EBNA1) may present few immunogenic targets, limiting CTL efficacy. Additionally, tumor microenvironmental suppression and immune escape mutations can diminish CTL persistence or function, underscoring the need for strategies to optimize antigen selection and enhance *in vivo* expansion of these cells.

### CAR-T

CAR-T cell therapy involves genetically modifying a patient’s or donor’s T-cells to express a synthetic chimeric antigen receptor (CAR) that recognizes specific tumor-associated surface antigens. Once infused, these engineered T-cells can identify and destroy tumor cells in a major histocompatibility complex (MHC)-independent manner, overcoming one of the key immune evasion mechanisms of EBV-infected cells.

CD19-directed CAR-T cells have already revolutionized the treatment of B-cell lymphomas and acute lymphoblastic leukemia, and their role in EBV-associated B-cell lymphomas parallels that seen in EBV-negative settings, such as CD19-positive diffuse large B-cell lymphoma (DLBCL) (61). However, the therapeutic potential of CAR-T cells extends further in EBV-driven disease. Novel CAR constructs are being developed to specifically target EBV-related antigens such as LMP1 and LMP2, which are selectively expressed on the surface of EBV-transformed cells, allowing for more direct and virus-specific tumor killing (62, 63).

In classical Hodgkin lymphoma, CD30-directed CAR-T cells have demonstrated encouraging early results, reflecting the high CD30 expression characteristic of this disease. These EBV-specific or tumor-specific CAR-T cell designs hold the promise of achieving highly selective cytotoxicity while minimizing off-target effects. Ongoing preclinical and early clinical studies are investigating strategies to improve CAR-T cell persistence, trafficking into tumor sites, and resistance to the immunosuppressive tumor microenvironment that characterizes many EBV-driven lymphomas.

### Epigenetic and molecular inhibitors

EBV latent proteins profoundly reprogram host cell signaling and epigenetic machinery, promoting survival, proliferation, and immune escape. Consequently, targeting these aberrant signaling cascades and epigenetic regulators provides a promising therapeutic avenue. One of the most critical pathways is NF-κB, which is constitutively activated by LMP1, leading to transcription of anti-apoptotic and proliferative genes. NF-κB inhibitors are therefore being explored to disrupt this axis and restore apoptotic sensitivity in EBV-driven tumors (64).

Other key survival pathways influenced by EBV include the PI3K/AKT/mTOR cascade, which promotes cell growth and metabolic adaptation. Inhibitors of these signaling components can suppress tumor cell proliferation and sensitize them to chemotherapy or immune-mediated killing. Epigenetic modifiers such as histone deacetylase (HDAC) inhibitors and EZH2 inhibitors are also under investigation, as they can reverse EBV-induced transcriptional silencing and restore expression of viral or tumor suppressor genes (65, 66).

Proteasome inhibitors like bortezomib may exert therapeutic benefit by indirectly inhibiting NF-κB activation and promoting accumulation of pro-apoptotic factors (67). These agents can also modulate antigen presentation, potentially enhancing tumor immunogenicity and complementing immunotherapeutic strategies. Together, these molecular inhibitors offer a multi-pronged approach to disrupt EBV-driven oncogenic signaling and restore normal cellular control mechanisms.

### Vaccine strategies

The development of effective EBV vaccines is considered a cornerstone of long-term prevention and control of EBV-associated malignancies. Two major approaches are being pursued: prophylactic and therapeutic vaccination.

Prophylactic vaccines aim to prevent primary EBV infection or block the virus’s entry into B-cells and epithelial cells. Early candidates have focused on the envelope glycoprotein gp350, which mediates viral attachment to the B-cell receptor CD21 (68, 69). Vaccines targeting gp350 have demonstrated some success in preventing infectious mononucleosis, a common manifestation of primary EBV infection. However, completely preventing EBV persistence and subsequent tumorigenesis remains challenging, necessitating the inclusion of additional viral targets and adjuvants to induce durable humoral and cellular immunity.

Therapeutic vaccines, on the other hand, are designed to elicit potent cytotoxic T-cell responses against EBV-infected malignant cells. These typically target latent antigens such as LMP1 and LMP2, which are consistently expressed in many EBV-associated tumors (68, 69). The objective is to boost the host immune system’s ability to recognize and eradicate established tumors while minimizing immune tolerance. Early-phase clinical trials are evaluating recombinant viral vector or peptide-based vaccines incorporating these antigens, with encouraging evidence of immunogenicity and occasional clinical responses. Continued optimization of antigen selection, delivery platforms, and combination with immune checkpoint blockade may further enhance the efficacy of therapeutic EBV vaccines in the future.

The use of CAR-T and EBV vaccines are still highly experimental with limited clinical applicability,

**Table 2** summarizes ongoing trials on therapeutics of EBV-associated lymphoma.

**Table 2:** Ongoing trials on EBV-associated lymphoma therapeutics.

Trial number/ID	Patient population	Investigational drug/therapy	Outcomes measured
NCT00002663	EBV^+^ PTLD patients refractory to rituximab and chemo	Tabelecleucel infusion (phase I)	Safety and efficacy (response rates)
NCT05011058	Adults (≥18 yrs) with relapsed/refractory EBV^+^ lymphomas (DLBCL, HL, PTLD, ENKTCL etc.)	Nanatinostat (class I HDAC inhibitor) + Valganciclovir	Phase II: efficacy (response rate), safety, progression-free survival
NCT01094405	Relapsed/refractory EBV^+^ lymphoid malignancies & lymphoproliferative disorders	HQK-1004 (HDAC inhibitor) + Valganciclovir	Phase II: response rate, safety
NCT02973113	Patients with relapsed/refractory EBV^+^ lymphoma	Nivolumab + EBV-specific T cell infusion	Safety (phase I/II), adverse events, early efficacy signs
NCI-2022-08324	Patients with EBV-associated lymphoma or malignancy (various)	Genetically-modified C7R-expressing EBV-specific T cells (phase I)	Safety, tolerability, optimal dose
(Baylor/ViGenCell trial)	Patients with relapsed/refractory or high-risk extranodal NK/T cell lymphoma, nasal type	Donor CTLs engineered to kill LMP1/2/BARF1/EBNA-1-expressing cells (Phase I/II)	Safety; durable remission rates, relapse-free survival

## Challenges and knowledge gaps

Despite significant progress in understanding EBV biology and its role in lymphomagenesis, several challenges and knowledge gaps persist, hindering optimal patient management and the development of more effective therapies. The vast clinical and biological heterogeneity of EBV-associated lymphomas presents a major challenge. Each subtype has distinct pathological features, latency programs, and clinical behaviors, requiring tailored diagnostic and therapeutic approaches (1). While advances have been made, comprehensive molecular profiling is not universally available, and the nuanced interplay between viral factors, host genetics, and microenvironment within each subtype is still being elucidated. This complexity makes it difficult to apply a single, unified therapeutic strategy. Currently, for many lymphoma types (e.g., cHL, BL), EBV status is not routinely used to guide first-line treatment decisions, despite its prognostic implications (28, 47). This is partly due to the fact that standard regimens are generally effective and the added benefit of EBV-specific approaches in the upfront setting is not yet fully established. Incorporating EBV status into treatment algorithms would require further robust clinical trial evidence demonstrating superior outcomes with EBV-guided therapies. Many clinical trials for lymphomas do not stratify patients based on EBV status or specifically enroll patients with EBV-associated subtypes. This results in a lack of high-level evidence for optimal management strategies for these distinct entities (33). There is a critical need for dedicated, prospective clinical trials focusing on specific EBV-associated lymphomas to evaluate novel agents and refine existing therapies. While quantitative PCR for EBV DNA is a valuable tool for diagnosis and monitoring, particularly in PTLD and ENKTL, there is a lack of widespread standardization across different laboratories regarding assay methodologies, cut-off values, and interpretation of results (43, 44). This limits the comparability of data across studies and clinical centers. Standardized assays and established guidelines for their use would significantly improve clinical utility. In many regions of the world, particularly in developing countries where the burden of certain EBV-associated lymphomas (e.g., endemic BL, ENKTL) is high, access to advanced diagnostic techniques (e.g., EBER ISH, quantitative EBV DNA PCR) and sophisticated treatments (e.g., ICIs, CAR-T) remains limited (29, 38). Addressing these disparities is crucial for improving outcomes globally.

## Inborn errors of immunity and EBV-associated lymphomas

Recent advances in the field of inborn errors of immunity (IEIs) have greatly enhanced the understanding of the interplay between host immune defects and EBV-driven lymphomagenesis. Virus-associated neoplasia, including EBV-associated lymphomas, can often represent the first clinical manifestation of underlying IEIs, particularly in pediatric and young adult patients (70, 71). Among these, X-linked lymphoproliferative disease (XLP) types 1 and 2, caused by mutations in *SH2D1A* and *XIAP* respectively, are classical examples in which defective cytotoxic T-cell and NK-cell responses to EBV result in uncontrolled B-cell proliferation and life-threatening lymphoproliferative disease (71, 72). Similarly, activated PI3Kδ syndrome (APDS), due to *PIK3CD* or *PIK3R1* mutations, and common variable immunodeficiency (CVID) have been associated with EBV-positive lymphomas, reflecting impaired immune regulation and defective viral clearance (73). Other disorders such as CTPS1 deficiency, MAGT1 deficiency, and CD27/CD70 axis defects further underscore how disruption of cytotoxic lymphocyte function predisposes to persistent EBV infection and malignant transformation (71, 74). Recognition of these conditions is crucial, as EBV-associated lymphoma arising at an unusually early age, within a family history of immune dysregulation, or accompanied by autoimmunity, hypogammaglobulinemia, or hemophagocytic episodes, should prompt evaluation for underlying IEI (75). Genetic diagnosis not only informs pathogenesis but also guides therapeutic decisions, since certain IEIs, notably XLP and other severe cytotoxic pathway defects, are amenable to curative hematopoietic stem cell transplantation (HSCT) (72, 75). Integrating systematic immunologic and genomic assessment into the diagnostic work-up of EBV-associated lymphomas therefore represents a critical step toward precision medicine and improved outcomes in these patients.

## Discussion

EBV-associated lymphomas represent a fascinating and challenging group of malignancies, underscoring the complex interplay between viral infection and host oncogenesis. The information presented herein highlights the significant diversity of these neoplasms, emphasizing how the specific viral latency programs, the host’s immune status, and co-factors like malaria or immunosuppression profoundly influence disease behavior and prognosis. While the histological subtype largely dictates current treatment paradigms, there is a rapidly growing recognition of the unique biological features conferred by EBV presence, which increasingly points towards the need for more targeted and personalized therapeutic approaches. **Table 3** summarizes the prognostic and predictive value of EBV in various EBV-associated lymphoid malignancies.

**Table 3:** Prognostic and predictive value of EBV in various EBV-associated lymphomas.

Lymphoma subtype	Prognostic value of EBV	Predictive value of EBV (therapeutic implication)
Classical Hodgkin lymphoma	EBV positivity is associated with better prognosis in young adults and children. In elderly, association with worse outcome is debated	EBV^+^ cHL may respond better to PD-1 inhibitors (e.g., nivolumab, pembrolizumab) due to immune evasion via LMP1/PD-L1 pathway
EBV^+^ diffuse large B-cell lymphoma	EBV^+^ DLBCL of elderly is associated with poor prognosis, particularly in Asian and Latin American cohorts	EBV^+^ status may identify candidates for immune checkpoint blockade or EBV-specific CTL therapy
Extra-nodal NK/T-cell lymphoma	EBV DNA load correlates with tumor burden, relapse risk and survival. High EBV DNA post-treatment is associated with poor prognosis	EBV DNA levels can be used for monitoring response and detecting relapse. Targeted with PD-1 inhibitors, EBV-CTLs
Primary Effusion Lymphoma	Typically, co-infected with HHV8 and EBV. EBV role less clear; EBV^+^ PEL may not differ in prognosis significantly	EBV-LMP1/2 expression provides rationale for EBV-specific immunotherapy
Endemic Burkitt lymphoma	EBV^+^ endemic BL has better prognosis compared to sporadic (EBV^-^); viral latency type I associated	May respond to EBV-targeted vaccines or CTLs though clinical application is limited
Post-transplant lymphoproliferative disorder	EBV^+^ PTLD generally has worse prognosis if unresponsive to rituximab. High viral load is associated with higher mortality	EBV status is highly predictive of response to rituximab, EBV-specific CTLs (e.g., tabelecleucel)
Angioimmunoblastic T-cell lymphoma	EBV often found in B immunoblasts, not T cells. EBV^+^ status correlates with worse prognosis	No specific predictive role yet; indirect value in immune profiling
Peripheral T-cell lymphoma, NOS	EBV^+^ cases tend to have worse overall survival and higher relapse rate	Possible future role for PD-1 blockade or EBV-CTLs
HIV-associated lymphomas (e.g., DLBCL, PEL)	EBV positivity common but prognostic impact unclear due to confounding HIV factors	EBV provides target for immunotherapeutic strategies, especially in refractory disease

The detailed understanding of viral oncogenes like LMP1 and their direct impact on host signaling pathways provides clear molecular targets for drug development. Similarly, the mechanisms by which EBV promotes immune evasion, such as through PD-L1 upregulation, have directly informed the successful application of immune checkpoint inhibitors in these diseases. The impressive responses seen with PD-1 blockade in cHL and ENKTL, for instance, validate the strategy of leveraging the unique immunobiology of EBV-driven tumors.

Furthermore, the success of adoptive immunotherapy with EBV-specific CTLs in PTLD offers a compelling paradigm for precision immune targeting that could potentially be extended to other EBV-associated malignancies. Challenges remain in translating these successes to all EBV-positive lymphomas, particularly those with more restricted latency programs or in immunocompetent hosts, where the tumor cells may be less immunogenic or the endogenous immune response more robust. Nevertheless, these advancements underscore the importance of leveraging the viral component as a distinct therapeutic vulnerability.

Future research efforts must focus on several key areas. Firstly, integrating comprehensive EBV characterization (latency type, viral load, specific oncogene expression) into routine diagnostic workups will be crucial for refined risk stratification and treatment selection. Secondly, dedicated, well-designed clinical trials specifically for EBV-associated lymphoma subtypes are essential to establish optimal, evidence-based treatment guidelines. This includes evaluating novel combinations of chemotherapy with targeted agents or immunotherapies, and exploring the potential of maintenance strategies or adjuvant therapies to prevent relapse. Thirdly, continued research into novel therapeutic targets, beyond the current ICIs, for instance, targeting upstream EBV-mediated signaling or epigenetic dysregulation, holds significant promise. Finally, the development of both prophylactic and therapeutic EBV vaccines remains a long-term goal that could dramatically alter the landscape of these diseases. Collaborative research efforts across various disciplines; virology, immunology, oncology, and epidemiology will be paramount to translating these scientific insights into improved clinical outcomes.

## Conclusion

Epstein-Barr virus remains a pivotal and multifaceted factor in the pathogenesis of a significant proportion of lymphoid malignancies. The past few decades have witnessed remarkable advancements in our understanding of EBV biology, its intricate latency programs, and the diverse mechanisms by which it contributes to cellular transformation and immune evasion. These scientific insights have directly opened new avenues for both novel diagnostics and innovative immunotherapies. As the field continues to progress, the more precise incorporation of EBV-specific biomarkers into clinical practice, alongside the strategic deployment of targeted therapies that exploit the unique viral vulnerabilities, holds immense potential to significantly improve patient outcomes, particularly in high-risk and immunocompromised populations. The journey from initial discovery of EBV in lymphoma to current precision medicine approaches exemplifies the power of basic science research informing clinical translation. Continued collaborative efforts across epidemiology, virology, oncology, and immunology are not merely beneficial but essential to further unravel the complexities of EBV-associated lymphomagenesis and to ultimately translate these profound scientific insights into more effective and personalized clinical practice worldwide.
